# Early Cardiac Catheterization in Children with Congenital Heart Disease on Postoperative Extracorporeal Membrane Oxygenation: Safety, Outcomes, and Clinical Management

**DOI:** 10.3390/diagnostics16091367

**Published:** 2026-04-30

**Authors:** Burcu Çevlik, Ahmet Saki Oğuz, Ali Nazım Güzelbağ, Demet Kangel, Kahraman Yakut, Muhammet Hamza Halil Toprak, Abdullah Erdem, İbrahim Cansaran Tanıdır, Ali Can Hatemi, Erkut Öztürk

**Affiliations:** 1 Department of Pediatric Cardiology, Health Sciences University Basaksehir Cam and Sakura City Hospital, Istanbul 34480, Turkey; ahmetsaki@gmail.com (A.S.O.); anguzelbag@gmail.com (A.N.G.); demetkangel@gmail.com (D.K.); kahramanyakut@gmail.com (K.Y.); muhammedhamzatoprak@hotmail.com (M.H.H.T.); drabdullaherdem@hotmail.com (A.E.); cansaran@yahoo.com (İ.C.T.); erkut_ozturk@yahoo.com (E.Ö.); 2 Department of Pediatric Cardiovascular Surgery, Health Sciences University Basaksehir Cam and Sakura City Hospital, Istanbul 34480, Turkey; alicanhatemi@gmail.com

**Keywords:** extracorporeal membrane oxygenation, cardiac catheterization, congenital heart disease, interventional procedures

## Abstract

**Background**: Extracorporeal membrane oxygenation (ECMO) is lifesaving in pediatric patients with respiratory and/or cardiovascular failure. Cardiac catheterization is an important diagnostic and therapeutic tool in patients with congenital heart disease supported by ECMO, allowing the assessment of residual lesions, hemodynamically significant anatomical abnormalities, and unexplained indications for ongoing ECMO support. The timing and clinical contribution of cardiac catheterization in these patients are still debated. **Objective**: This study aimed to evaluate the indications, safety, and impact of cardiac catheterization on clinical management in pediatric patients receiving postoperative ECMO support. **Methods**: This single-center, retrospective study examined 39 pediatric patients under the age of 18 who underwent postoperative cardiac catheterization with ECMO support between January 2022 and December 2025. Demographic data, procedure characteristics, and clinical outcomes were analyzed. **Results**: Of the 190 patients under postoperative ECMO support, 39 underwent catheterization. The median age of the patients was 2.5 months (range, 6 days–180 months) and median weight was 4.2 kg (range, 2.8–57 kg). The most frequent diagnoses were ventricular septal defect-pulmonary atresia (VSD-PA) in 20.5% (*n* = 8) and transposition of the great arteries (TGA) in 15.3% (*n* = 6). The indication for catheterization was to investigate the reason for ECMO placement in 26 patients (66.6%). Most patients underwent catheterization within the first 24 h after ECMO initiation. Patients who underwent catheterization represented a higher-risk subgroup, with a greater proportion of STAT 4-5 procedures (59% vs. 40%) compared with the overall ECMO cohort. Cardiac catheterization resulted in a change in clinical management in 25.6% of patients through catheter-based intervention or surgical revision. Survival in the catheterized subgroup was 12.8%, reflecting the high-risk nature of this population. **Conclusions**: Cardiac catheterization in pediatric patients on postoperative ECMO support can be performed with a low complication rate and can significantly alter clinical management. Cardiac catheterization should be considered an important diagnostic and therapeutic modality, particularly in the presence of suspected residual lesions or unexplained hemodynamic instability. Additionally, we recommend that cardiac catheterization be performed promptly within the first 24–48 h in this patient group on ECMO support.

## 1. Introduction

Extracorporeal membrane oxygenation is a life-support modality used for the management of severe respiratory and/or cardiovascular failure, including preoperative hemodynamic stabilization in patients with congenital heart disease (CHD), medically refractory fatal arrhythmias, unexplained sudden cardiac arrest, and low cardiac output syndrome following cardiac surgery [1,2,3,4,5]. In recent years, ECMO support has become a standard treatment method in the management of respiratory and heart failure in both pediatric and adult patients [1,2]. The outcomes of ECMO support vary depending on the underlying cause. As seen in the Extracorporeal Life Support Organization’s registry analysis, patients with congenital heart disease who require ECMO support have a higher hospital mortality rate [3].

In patients requiring ECMO after heart surgery, the presence of residual lesions has been identified as a potential cause of mortality. Addressing these residual lesions is crucial. Non-invasive methods such as transthoracic echocardiography (TTE), cardiac magnetic resonance imaging (C-MR), and cardiac computed tomography (CTA) angiography may not be sufficient for this purpose in some cases. Cardiac catheterization plays a critical role in identifying hemodynamically significant anatomical abnormalities and determining the underlying causes of persistent ECMO dependence. In addition, it provides the opportunity to correct residual lesions interventionally in some cases [6,7]. In this context, non-invasive imaging modalities may be limited in ECMO-supported patients due to technical and clinical challenges [8]. There are studies reporting that catheterization can reduce the duration of ECMO, but the data are limited [9,10].

This study aimed to evaluate the use of early cardiac catheterization, the interventions performed, and the safety of these procedures in pediatric patients supported by ECMO.

## 2. Materials and Methods

This study was designed as a single-center retrospective study and conducted in accordance with the principles of the Declaration of Helsinki. The study was approved by the Ethics Committee of Istanbul Çam and Sakura City Hospital, Health Sciences University of Turkey (Protocol Code: 2025/337 and Date: 10 December 2025). Informed consent was obtained from the relatives of the patients.

Between January 2022 and December 2025, 190 cases that received ECMO support at our clinic were screened. Pediatric patients (<18 years) who required venoarterial ECMO support following congenital heart surgery and subsequently underwent cardiac catheterization were included. Patients were classified according to the Thoracic Surgeons-European Society of Cardio-Thoracic Surgery (STAT) score [11].

All patients were evaluated with TTE during the diagnosis and follow-up process. Patients whose TTE could not be adequately assessed under ECMO support, whose hemodynamics were unstable despite ECMO support, or who had suspected residual defects on echocardiography were evaluated with cardiac catheterization.

The ECMO circuit consisted of a Sorin Revolution, Lilliput 2 PMP oxygenator (Sorin Group Italia, Mirandola, Italy) and a modified centrifuge pump for both arterial and venous patients. Venoarterial ECMO was performed in all patients. ECMO pump flow was started at 150 mL/kg/min and changed after maintaining end-organ perfusion, an increase in systemic venous saturation, and a decrease in lactic acidosis.

Echocardiographic evaluation was performed using a Philips EPIQ CVx Cardiac Ultrasound (Philips, Amsterdam, The Netherlands). Parasternal (long and short axis), apical (four and five chambers), subcostal, and suprasternal sections were obtained. Cardiac morphology was assessed using a segmental approach. The main components of this approach included atrial situs, venoatrial junction (systemic and pulmonary venous return), atrium-ventricular junctions, ventricles, ventricular-great artery junction, intracardiac defects, and extracardiac vascular anomalies. The examinations were performed by two pediatric cardiologists experienced in the field of congenital heart disease.

Cardiac catheterization procedures were performed under general anesthesia via jugular and femoral access using a biplane angiography system (Philips^®^ AZURION 7 B12, Philips). Patients were adequately hydrated before and after the procedure, and renal function tests were performed before and after each procedure. Hemodynamic data, including intracardiac pressures and oxygen saturation levels, were recorded from multiple heart chambers, the aorta, and vena cavae using fluid-filled catheters. The patient was weaned from short-term ECMO support under the supervision of the perfusion team while hemodynamic data were being measured. During angiographic evaluation, an intravenous iodinated contrast medium (Kopaq 350 mg/mL) was used at a dose not exceeding 4 mL/kg. Contrast injections were performed into diagnostically appropriate cardiac chambers to assess the presence of residual defects. All procedures were performed by an experienced pediatric cardiology team. Interventional procedures were performed on residual lesions when necessary.

## 3. Statistical Analysis

Descriptive and inferential statistical methods were used for data analysis. The distribution of continuous variables was assessed using Kolmogorov–Smirnov and Shapiro–Wilk tests. Variables showing a normal distribution were analyzed using parametric tests, while variables not showing a normal distribution were analyzed using non-parametric tests. Categorical variables were presented as numbers and percentages. Anthropometric measurements were analyzed using percentages and standard deviation scores (z-score). Associations between categorical variables were evaluated using the Chi-square test. All statistical analyses were performed using SPSS version 15.0 (SPSS Inc., Chicago, IL, USA), and a *p*-value < 0.05 was considered statistically significant.

## 4. Results

Between January 2022 and December 2025, a total of 190 patients required venoarterial ECMO support. Of these 190 patients, 39 (20.5%) underwent diagnostic or interventional cardiac catheterization. Twenty patients were female. Their median age was 2.5 months (range, 6 days–180 months), median weight was 4.2 kg (range, 2.8–57 kg), and median height was 54 cm (range, 47–158 cm). Demographic data of the patients are summarized in Table 1.

The most frequent diagnoses were VSD-PA (20.5%, *n* = 8) and TGA (15.3%, *n* = 6) (Table 1). Twenty-seven patients had double ventricle physiology. Patients who underwent catheterization represented a higher-risk subgroup with a higher rate of STAT 4-5 procedures (59% vs. 40%) compared to the general ECMO cohort (*p* < 0.001) (Table 2). One patient had Down syndrome and one had VACTERL association.

All patients were placed on ECMO support related to the operation. Indications for extracorporeal membrane oxygenation were hemodynamic instability in 34 patients, failure to terminate intraoperative bypass in four patients, and cardiac arrest in one patient (while intubated in the intensive care unit and receiving high-dose inotropic support). The indications for catheterization were investigating the reason for ECMO placement in 26 patients (66.6%), postoperative coronary evaluation in 9 patients, investigating the cause of desaturation in two patients, need for BAS and ductus arteriosus stent after echocardiographic evaluation in one patient, and prolonged ECMO duration in one patient. Our reasons for preferring catheterization over cardiac computed tomography angiography to investigate the cause of ECMO in these patients were hemodynamic instability, risks associated with patient transport, and the therapeutic role of cardiac catheterization.

**Figure 1:**
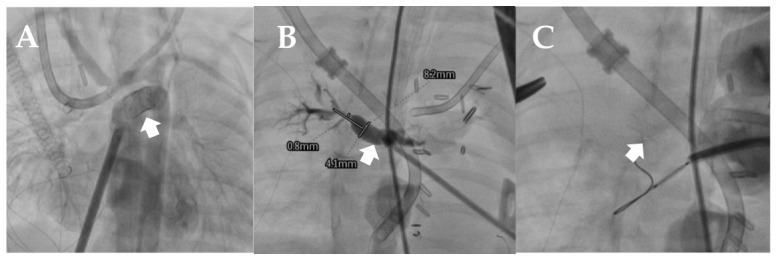
(**A**) Ductus arteriosus stent and BAS procedure in a patient with HLHS. (**B**,**C**): MAPCA stent placement and post-stent injection in a patient with VSD-PA.

**Figure 2:**
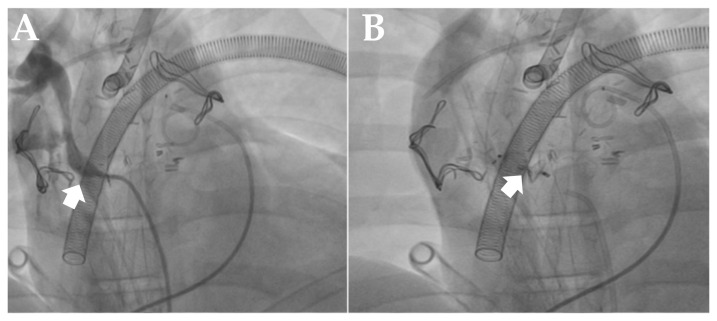

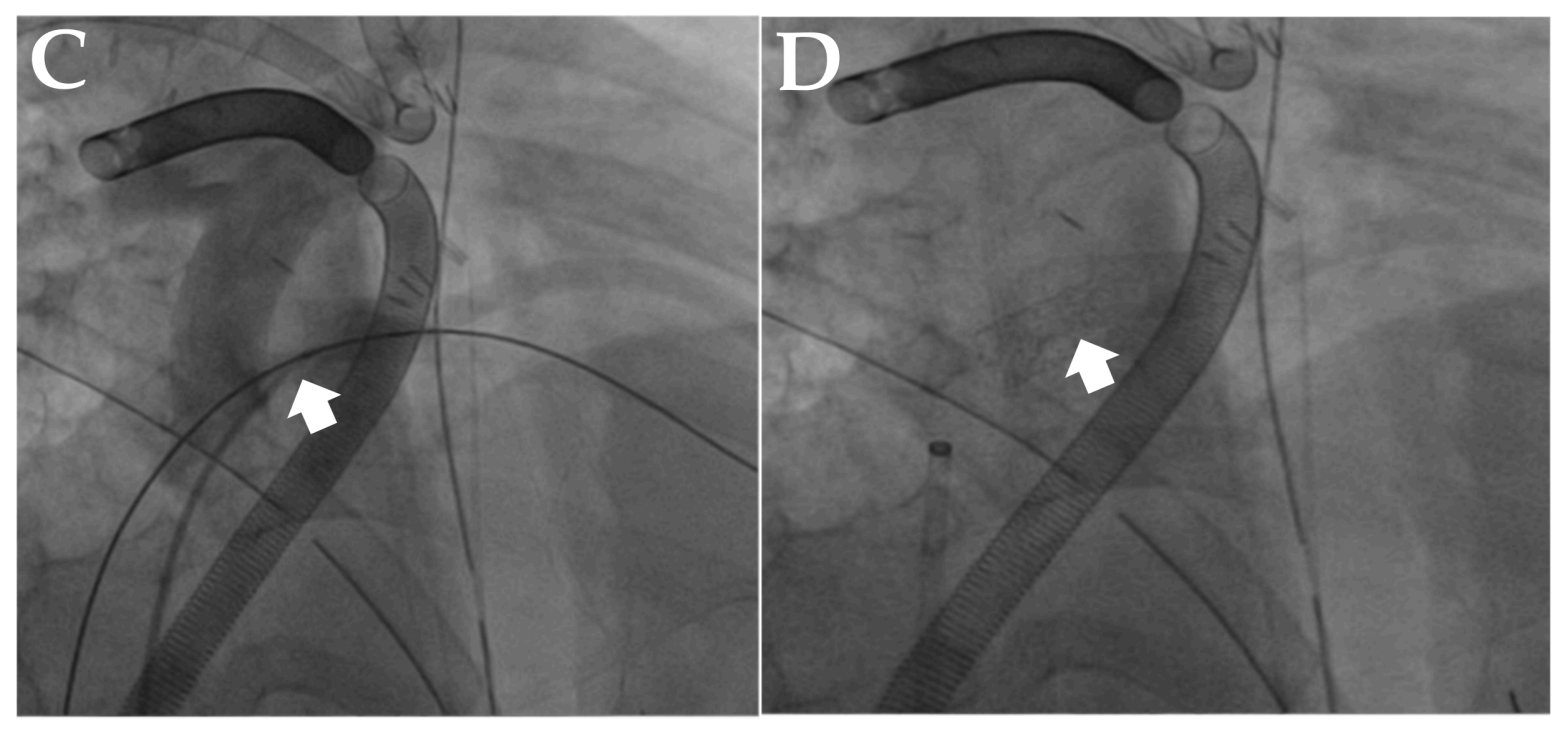
(**A**,**B**): MAPCA image and post-closure injection in a patient diagnosed with VSD-PA who underwent MAPCA closure. (**C**,**D**): Fontan fenestration stenosis and post-stent placement image in a patient diagnosed with cTGA who underwent Fontan surgery.

**Figure 3:**
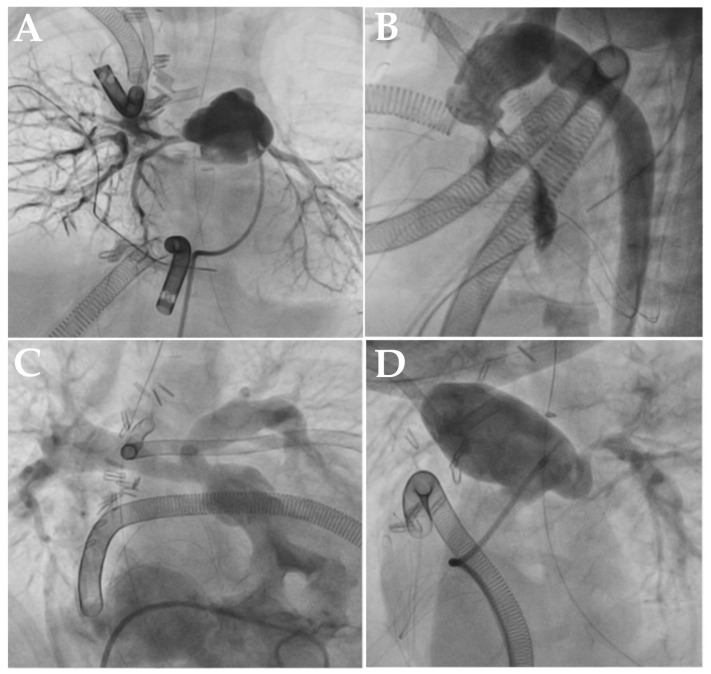
(**A**) Image showing lack of blood supply to the right pulmonary artery (RPA) during postoperative catheterization of a patient with VSD-PA who underwent unifocalization. (**B**) Image showing left anterior descending (LAD) filling defect during catheterization of a patient with arterial switch (ASO) who underwent total repair and Taussig-Bing who underwent arch reconstruction. (**C**) Image showing double right ventricular outflow tract (RVOT) and stenosis in the peripheral pulmonary arteries during catheterization of a patient with DOBV who underwent total repair. (**D**) Image showing an aneurysmal patch placed in the main pulmonary artery (MPA) and stenosis in the LPA during catheterization of a patient with TOF who underwent total repair.

The median time from initiation of ECMO support to catheterization was 1 day (range, 0–5 days). Thirty-three patients underwent catheterization within the first 24 h, two patients within 24–48 h, and four patients after 48 h. Interventional procedures were performed during catheterization in 12.8% of patients; one patient underwent stent placement in the stenosis of the fenestration of a Fontan operation, one patient underwent left pulmonary artery (LPA) stent placement, one patient underwent MAPCA stent placement, one patient underwent BAS and ductus arteriosus stent placement, and one patient underwent MAPCA closure (Table 3, Figure 1 and Figure 2). 12.8% (*n* = 5) of the cases were reoperated on after catheterization (Table 4 and Figure 3).

The median pre-procedure saturation was 95% (range, 65–100%) and the median post-procedure saturation was 96% (range, 75–100%). In catheterization procedures, the median procedure time was 25 min (range, 10–135 min), the median fluoroscopy time was 264 s (range, 84–864 s), the median radiation dose was 398 mGy (range, 103–952 mGy) and 3226 cGycm2 (range, 1076–8900 cGycm2), and the median contrast agent used was 25 mL (range, 5–100 mL) (Table 1). In patients undergoing interventional procedures, the rate of contrast agent use (*p* = 0.007), procedure time (*p* = 0.009), and fluoroscopy time (*p* = 0.02) were found to be higher.

During catheterization, minor complications occurred in 3 cases (7.6%). In one case, bradycardia developed when disconnected from ECMO for pressure measurement reliability during the procedure and was immediately stabilized by restoring ECMO support. In the case where ductus arteriosus stenting was performed, atrioventricular block causing hypotension developed and returned to sinus rhythm with increased ECMO flow rate, and in another case, self-limiting pulmonary hemorrhage developed during LPA stenting.

Cardiac catheterization resulted in a change in clinical management in 25.6% of patients through catheter-based intervention or subsequent surgical revision. This finding suggests that catheterization is selectively applied in high-risk and complex patients (those with high STAT 4-5 scores). In long-term follow-up after catheterization, two patients died due to severe pulmonary hemorrhage, and the remaining cases died due to multiorgan failure.

## 5. Discussion

In this single-center retrospective study, we evaluated the indications, safety, and clinical impact of cardiac catheterization in patients receiving ECMO support. The study was conducted in a tertiary cardiac surgery center where ECMO support is commonly used. By evaluating the outcomes of thirty-nine patients, we aimed to highlight the importance of catheterization in ECMO-supported patients, an area with a limited number of studies. The low survival rate observed in the catheterized subgroup likely reflects the selection of patients with more complex anatomy and higher operative risk rather than a negative impact of the procedure itself. Our findings support the concept that early catheterization may facilitate the rapid identification of residual lesions and guide timely surgical or catheter-based interventions in ECMO-supported patients.

Extracorporeal membrane oxygenation support is now widely used to treat cardiac surgery patients with conditions such as recurrent cardiac arrest requiring continuous resuscitation, low cardiac output despite adequate inotropic support, fulminant myocarditis, uncontrolled arrhythmias, and pre- and post-transplantation support requirements, and has become a part of standard treatment [1,2,3,12,13,14].

The use of TTE in patients under ECMO support is limited due to poor acoustic windows or delayed sternum closure, which is frequently encountered in the early postoperative period [8,15]. When other non-invasive imaging methods are insufficient, it is important to perform interventional evaluation under ECMO to identify residual lesions and hemodynamically significant anatomical problems and to assess the need for ECMO support for reasons that are not understood [16,17,18,19].

Rodas et al. [20] reported that they performed cardiac catheterization on 36% (51/140) of patients on ECMO support, and Both et al. [3] on 28% (54/192). Bahaidarah et al. reported that the rate of postoperative patients under ECMO support who underwent catheterization was 40.7% [21]. In our study, this rate was slightly lower than in the literature, at 20.5%. Rodas et al. reported that 92% of patients evaluated with catheterization had single ventricle physiology, and Kelsey et al. [22] reported that 31.6% had single ventricle physiology. In our study, 30.7% of our patients had single ventricle physiology.

Previous studies have reported that the most common indications for cardiac catheterization in patients receiving ECMO support are failure to wean from ECMO and persistent desaturation [21,23]. In our study, the most common reason (66.7%) was to investigate the reason for needing ECMO support.

Panda et al. reported a series of 22 out of 59 patients on ECMO who underwent cardiac catheterization from February 2009 to August 2012. They discovered problems in seventeen of their patients that necessitated further therapeutic procedures and showed that seven patients required catheter-based intervention, eight required surgical intervention, and two required hybrid intervention [24]. In our study, 12.8% of our cases underwent interventional procedures during catheterization, and 12.8% required reoperation after catheterization. All these patients underwent catheterization within the first 24 h, and 30% of these ten patients survived.

Rodas et al. [20] performed interventional procedures in 64% of patients, mostly PA stenting and BAS, while McLean et al. [22] performed pulmonary artery re-intervention most frequently. In our study, 12.8% (*n* = 5) of patients underwent interventional procedures, and similarly, the rate of pulmonary artery re-intervention was higher. We also found that cardiac catheterization led to a change in clinical management in 25.6% of patients through catheter-based intervention and subsequent surgical revision.

In the study by Rodas et al. [20], the median time from ECMO cannulation to catheterization was 1.25 days. In the study by Kato et al. [25], this time was 1 day (0–11 days), and similarly, in our study, the time from ECMO support to catheterization was 1 day (0–5 days), and all patients except four underwent the procedure within the first 48 h. After catheterization, 25.6% of patients required re-intervention, and 30% of them survived. We believe that in these patients under ECMO, if there is an unexplained condition, catheterization within the first 24–48 h without much delay is important.

Rodas et al. [20] reported that the complication rate during catheterization was 3% and Bergersen et al. [26] reported that it was 16%, while McLean et al. [22] reported that the most common complications were arrhythmia (3.3%), bleeding events (2.7%), unplanned cardiac surgery (1.8%), and other unplanned operations (1.8%). Previous studies have demonstrated that cardiac catheterization can be safely performed in children receiving ECMO support, with acceptable complication rates [27]. In our study, like the literature, the complication rate during catheterization was 7.6%, and these were minor complications.

Survival rates in ECMO patients undergoing cardiac catheterization have been shown to range from 29 to 74%; however, available data suggest that early catheterization may improve outcomes in patients under ECMO [21,28]. These studies are mostly single-center studies conducted in the early stages. Desjardins et al. reported a lower survival rate (overall survival 14%) [29]. In our study, catheterization after ECMO was performed early in almost all patients, and we did not have the opportunity to compare it with late-stage catheterization.

In our study, survival was 12.8% in cases requiring catheterization under ECMO support. Survival was lower in the catheterized subgroup; it had a significantly higher rate of STAT 4-5 procedures (59% vs. 40%), indicating a significantly higher operative risk profile. This finding suggested that it was related to the indication and disease severity rather than a negative effect of catheterization. Sixty percent of the surviving patients underwent an interventional procedure during catheterization or reoperation after catheterization.

The main limitations of our study were its single-center, retrospective nature and limited sample size. The fact that most of our patients underwent catheterization early prevented a temporal comparison analysis with patients who underwent catheterization late. The homogeneous distribution of our patient group, all of whom had congenital heart disease and underwent surgery, can be considered an advantage. This study presents a cohort of congenital heart surgery patients demonstrating that catheterization can be safely applied in high-risk postoperative ECMO patients and leads to a significant change in management. Larger, multicenter, prospective studies will more clearly reveal the impact of catheterization timing and patient selection on clinical outcomes.

## 6. Conclusions

Cardiac catheterization can be safely performed in selected pediatric patients receiving ECMO support after congenital heart surgery, with a low rate of procedure-related complications. In our cohort, early catheterization—most frequently performed within the first 24 h—allowed identification of clinically significant residual lesions and resulted in a change in clinical management in 25.6% of patients through catheter-based interventions or subsequent surgical revision. Despite the overall low survival rate reflecting the high-risk nature of this population, our findings suggest that early catheterization plays a crucial role in guiding timely decision-making in ECMO-supported patients with unexplained hemodynamic instability. Therefore, cardiac catheterization should be considered an essential diagnostic and therapeutic tool, particularly within the first 24–48 h of ECMO support in appropriately selected patients.

Future prospective multicenter studies are needed to better define optimal patient selection and timing of catheterization in this high-risk population.

## Figures and Tables

**Table 1:** Demographic characteristics of the patients.

Patient Characteristics	*n*	%	Median Range
**Sex**			
**Male**	19	48.8	
**Female**	20	51.2	
**Median age (month)**			2.5 (0.2–180)
**Median body height (cm)**			54 (47–158)
**Median body weight (kg)**			4.2 (2.8–57)
**Diagnosis**			
**VSD-PA**	8	20.5	
**TGA**	6	15.3	
**TOF**	4	10.2	
**HLHS**	3	7.7	
**DORV-PA**	3	7.7	
**CAVSD**	3	7.7	
**Taussig-Bing**	2	5.1	
**cTGA**	2	5.1	
**Trunkus arteriozus**	1	2.5	
**IVS-PA**	1	2.5	
**Aortic stenosis**	1	2.5	
**DOBV**	1	2.5	
**İnterrupted aortic arch**	1	2.5	
**DORV**	1	2.5	
**TGA-VSD**	1	2.5	
**TA-VA discordance**	1	2.5	
**STAT scores**			
**STAT 2**	5	12.8	
**STAT 3**	10	25.6	
**STAT 4**	11	28.2	
**STAT 5**	13	33.3	
**Median ECMO time (day)**			6 (3–26)
**Median ECMO-catheterization duration (day)**			1 (0–5)
**Indication for catheterization**			
**Investigating the reason for ECMO**	26	66.7	
**Postop coronary evaluation**	9	23.2	
**Desaturation**	2	5.1	
**Need for interventional procedure**	1	2.5	
**Prolonged ECMO duration**	1	2.5	
**Median prodecure time (m)**			25 (10–135)
**Median fluoroscopy time (s)**			264 (84–864)
**Median radiation dose (mGy)**			398 (103–952)
**Median radiation dose (cGYcm^2^)**			3226 (1076–8900)
**Median amount of contrast agent (cc)**			25 (5–100)
**Presence of complications**	3	7.6	

**Abbreviations:** CAVSD, Complete atrioventricular septal defect; cTGA, Corrected TGA; DOBV, Double outlet both ventricles; DORV-PA, Double outlet right ventricle-pulmonary atresia; HLHS, Hypoplastic left heart syndrome; IVS-PA, Pulmonary atresia with intact ventricular septum; TA-VA discordance, Tricuspid atresia-ventriculoarterial discordance; TOF, Tetralogy of Fallot.

**Table 2:** Distribution of STAT Mortality Categories and Survival in the Overall Postoperative ECMO Cohort and the Catheterized Subgroup.

STAT Category	Overall ECMO Cohort (*n* = 190)			Catheterized Subgroup (*n* = 39)		
	Total *n*	Survivors *n* (%)	Deaths *n* (%)	Total *n*	Survivors *n* (%)	Deaths *n* (%)
**STAT 4-5**	76	24 (31.6%)	52 (68.4%)	23	3 (13%)	20 (87%)
**STAT 1-3**	114	55 (48.2%)	59 (51.8%)	16	2 (12.5%)	14 (87.5%)
**Total**	190	79 (41.6%)	111 (58.4%)	39	5 (12.8%)	34 (87.2%)

STAT: Society of Thoracic Surgeons-European Association for Cardio-Thoracic Surgery Congenital Heart Surgery Mortality Categories (STAT 4-5 proportion: *p* = 0.01, survival comparison: *p* < 0.001) (*p* values were calculated using the Chi-square test).

**Table 3:** Characteristics of patients undergoing interventional procedures during catheterization while on ECMO support.

	1	2	3	4	5
**Age**	15 d	3 m	4 m	186 m	129 m
**Diagnosis**	HLHS	VSD-PA	DORV-PA	VSD-PA	cTGA-VSD-PS
**Operation**	No	unifocalization and central shunt	BT shunt	unifocalization	Fontan operation
**ECMO time**	Postop day 1	Postop day 2	Postop day 2	Postop day 1	Postop day 2
**ECMO-catheterization duration**	1 day	1 day	1 day	1 day	1 day
**İndication for catheterization**	Need for DA stenting and BAS	Investigating the reason for ECMO	Investigating the reason for ECMO	Investigating the reason for ECMO	Investigating the reason for ECMO
**Interventional procedure**	DA stenting and BAS	MAPCA stenting	LPA stenting	MAPCA closure	Fontan fenestration stenting
**Pre-treatment sat (%)**	98	95	65	98	82
**Post-treatment sat (%)**	98	95	80	99	94
**Procedure time (m)**	30	135	120	25	30
**Fluoroscopy time (s)**	504	864	362	330	464
**Radiation dose (cGycm^2^)**	1886	5406	1950	7300	4950
**Radiation dose (mGy)**	170	857	158	897	505
**Contrast agent (mL)**	20	50	20	100	50
**Complication**	Bradycardia	No	No	No	No
**Mortality and reason**	Yes (multiorgan failure)	Yes (multiorgan failure)	No	Yes (multiorgan failure)	Yes (pulmonary hemorrhage)

**Table 4:** Characteristics of patients on ECMO support who underwent reoperation after catheterization.

	1	2	3	4	5
**Age (m)**	4	8	0.3	0.2	5.5
**Diagnosis**	VSD-PA	TOF	Taussig-Bing	DOBV	TOF
**Comorbidity**	No	VACTERL	No	No	No
**Operations**	Unifokalization and RV-PA conduit	Total repair	Arterial switch and arch reconstruction	Total repair	Total repair
**ECMO time**	Postop day 1	Postop day 1	Postop day 2	Postop day 2	Postop day 1
**ECMO-catheterization duration**	5 days	1 day	1 day	2 days	1 day
**İndication for catheterization**	Extension of ECMO duration	Coronary evaluation	Coronary evaluation	Coronary evaluation	Investigating the reason for ECMO
**Diagnosis made by catheterization**	Lack of filling in RPA	Stenosis in LPA	Coronary artery filling was reduced	Patch-derived double RVOT and stenosis in the peripheral pulmonary arteries	The patch placed in the MPA was aneurysmal and quite large, and the peripheral pulmonary arteries were hypoplastic
**Procedure time (m)**	25	20	38	45	30
**Fluoroscopy time (s)**	354	284	114	240	360
**Radiation dose (cGycm^2^)**	5280	8900	2172	5983	2247
**Radiation dose (mGy)**	472	715	214	592	336
**Contrast agent (mL)**	35	45	20	30	30
**Complication**	No	No	No	No	No
**Reoperation**	MAPCA expansion	LPA reconstruction	LAD bypass	Reconstruction of the pulmonary artery and its branches	Reconstruction of the pulmonary artery and its branches
**Exitus and reason**	Ex (multiorgan failure)	Alive	Ex (multiorgan failure)	Alive	Ex (multiorgan failure)

**Abbreviations:** LAD, Left anterior descending artery; LPA, left pulmonary artery. RPA, right pulmonary artery; VACTERL, (V = vertebral anomalies; A = anal atresia; C = cardiac (heart) defects; T = tracheal anomalies including tracheoesophageal (TE) fistula; E = esophageal atresia; R = renal (kidney) anomalies and radial dysplasia (thumb/radial side of the limb); L = other limb abnormalities).

## Data Availability

The data presented in this study are available on request from the corresponding author due to privacy restrictions.
